# Contemporary management of patients with atrial fibrillation in the Netherlands and Belgium: a report from the EORP-AF long-term general registry

**DOI:** 10.1007/s12471-021-01634-y

**Published:** 2021-09-15

**Authors:** Ö. Erküner, M. van Eck, O. Xhaet, H. Verheij, J. Neefs, A. Duygun, R. Nijmeijer, S. A. M. Saïd, H. Uiterwaal, V. Hagens, R. Bhagwandien, T. Szili-Torok, N. Bijsterveld, G. Tjeerdsma, J. Vijgen, A. Friart, E. Hoffer, P. Evrard, M. Srynger, J. Meeder, J. R. de Groot, J. van Opstal, R. Gevers, G. Y. H. Lip, G. Boriani, H. J. G. M. Crijns, J. G. L. M. Luermans, G. H. Mairesse

**Affiliations:** 1 Department of Cardiology, Maastricht University Medical Center + (MUMC+), Maastricht, The Netherlands; 2 Cardiovascular Research Institute Maastricht (CARIM), Maastricht University, Maastricht, The Netherlands; 3 Department of Cardiology, Jeroen Bosch Hospital, ’s Hertogenbosch, The Netherlands; 4 Department of Cardiology, CHU Namur, Yvoir, Belgium; 5 Department of Cardiology, Medisch Spectrum Twente, Enschede, The Netherlands; 6 Department of Cardiology, Academic Medical Centre Amsterdam, Amsterdam, The Netherlands; 7 Department of Cardiology, VieCuri Medical Center, Venlo, The Netherlands; 8 Department of Cardiology, Tjongerschans Hospital Heerenveen, Heerenveen, The Netherlands; 9 Department of Cardiology, Ziekenhuis Groep Twente, Hengelo, The Netherlands; 10 Department of Cardiology, Flevo Hospital, Almere, The Netherlands; 11 Department of Cardiology, Ommelander Hospital Group, Delfzijl, The Netherlands; 12 Department of Cardiology, Erasmus Medical Center, Rotterdam, The Netherlands; 13 Department of Cardiology, Jessa Hospital, Hasselt, Belgium; 14 Department of Cardiology, CHU Tivoli, La Louvière, Belgium; 15 Department of Cardiology, CHR Citadelle, Liège, Belgium; 16 Department of Cardiology, CHC St Joseph, Liège, Belgium; 17 Department of Cardiology, CHU Liège, Liège, Belgium; 18 Institute of Cardiovascular Sciences, University of Birmingham, Birmingham, UK; 19 Aalborg Thrombosis Research Unit, Department of Clinical Medicine, Aalborg University, Aalborg, Denmark; 20 Modena University Hospital, Department of Cardiology, University of Modena and Reggio Emilia, Modena, Italy; 21 Arlon and Clinique Ste Thérèse, Department of Cardiology, Cliniques du Sud-Luxembourg, Bastogne, Belgium

**Keywords:** Atrial fibrillation, Undertreatment, Overtreatment, Anticoagulant, EORP, Registry

## Abstract

**Background:**

Contemporary data regarding the characteristics, treatment and outcomes of patients with atrial fibrillation (AF) are needed. We aimed to assess these data and guideline adherence in the EURObservational Research Programme on Atrial Fibrillation (EORP-AF) long-term general registry.

**Methods:**

We analysed 967 patients from the EORP-AF long-term general registry included in the Netherlands and Belgium from 2013 to 2016. Baseline and 1‑year follow-up data were gathered.

**Results:**

At baseline, 887 patients (92%) received anticoagulant treatment. In 88 (10%) of these patients, no indication for chronic anticoagulant treatment was present. A rhythm intervention was performed or planned in 52 of these patients, meaning that the remaining 36 (41%) were anticoagulated without indication. Forty patients were not anticoagulated, even though they had an indication for chronic anticoagulation. Additionally, 63 of the 371 patients (17%) treated with a non-vitamin K antagonist oral anticoagulant (NOAC) were incorrectly dosed. In total, 50 patients (5%) were overtreated and 89 patients (9%) were undertreated. However, the occurrence of major adverse cardiac and cerebrovascular events (MACCE) was still low with 4.2% (37 patients).

**Conclusions:**

Overtreatment and undertreatment with anticoagulants are still observable in 14% of this contemporary, West-European AF population. Still, MACCE occurred in only 4% of the patients after 1 year of follow-up.

**Supplementary Information:**

The online version of this article (10.1007/s12471-021-01634-y) contains supplementary material, which is available to authorized users.

## What’s new?

- The majority of patients with atrial fibrillation (AF) who are on anticoagulation are still on the ‘older’ vitamin K antagonists
- Even though the Netherlands and Belgium are, in general, comparable countries, striking differences in the management of AF patients are observed
- Overtreatment and undertreatment with anticoagulants are still observable in a considerable proportion of West-European AF patients

## Introduction

Atrial fibrillation (AF) is the most common cardiac arrhythmia worldwide. In 2010, the prevalence of AF in the European Union was estimated at 8.8 million individuals. By 2060, this number will likely more than double to 17.9 million individuals [1]. In addition to this widespread occurrence of AF, the arrhythmia is associated with morbidities as well as with complications, the most important being ischaemic stroke, making it a major public health problem with inherent extensive economic burden [2].

Registries such as the one described in this paper, the EURObservational Research Programme on Atrial Fibrillation long-term general (EORP-AF LTG) registry, are important means to assess the characteristics, treatment, and outcomes of contemporary AF patients. Furthermore, guideline adherence can be checked and awareness raised to improve the treatment of AF patients. Therefore, we aimed to analyse these aspects in the patients from the EORP-AF LTG registry included in the Netherlands and Belgium, focusing on comorbidities at baseline, guideline adherence in treatment of AF, progression of AF, and occurrence of major adverse cardiac and cerebrovascular events (MACCE) after 1 year.

## Methods

The EORP-AF LTG registry is initiated by the European Society of Cardiology, conducted in 27 countries, including a total of 11,096 patients from 2013 to 2016 across 250 centres and it is the successor of the EORP-AF Pilot General Registry [3, 4]. For this analysis, we included all patients from the Netherlands and Belgium included in the registry. The study was approved by the Institutional Review Boards of all including centres in Belgium and the Netherlands. All patients provided written informed consent. Patient baseline data were obtained and the patients were followed for two consecutive years. The exact methods have been published previously [3]. We describe the baseline characteristics and 1‑year follow-up data of the patients from the Netherlands and Belgium.

MACCE was defined as a composite of cardiovascular death, ischaemic stroke/transient ischaemic attack (TIA), systemic thromboembolism, myocardial infarction, and major bleeding (intracranial bleeding, bleeding requiring hospitalisation or lengthening of stay, causing a haemoglobin level drop of > 2 g/l, or requiring blood transfusion). AF progression was defined as paroxysmal AF at baseline becoming persistent/permanent AF at follow-up, whereas AF regression was defined as persistent/permanent AF at baseline becoming paroxysmal AF at follow-up. For this definition of AF progression, patients classified as first diagnosed AF at baseline were reclassified if possible, i.e. if patients had spontaneous or pharmacological conversion at baseline, they were reclassified as paroxysmal AF. Failed attempt of cardioversion was classified as persistent AF. All others were classified as unknown, including those with successful electrical cardioversion. The estimated glomerular filtration rate (eGFR) was calculated with the CKD-EPI (chronic kidney disease epidemiology) formula, using serum creatinine concentration, gender, age, and race [5].

### Statistical analysis

Data were analysed with SPSS statistical software (version 23.0, SPSS Inc., IBM Corp., Armonk, NY, USA). Continuous variables are reported as mean ± standard deviation if normally distributed and as median and interquartile range if not. Normally distributed continuous variables were compared between groups using the independent samples t‑test, whereas not normally distributed continuous variables were compared using the Mann-Whitney U test. Categorical variables are reported as observed number of patients and percentage. Among-group comparisons were made using a χ^2^ test. Fisher’s exact test was used in case of any expected cell count < 5.

## Results

In total, 967 consecutive patients were included across 23 centres, 648 (67%) from the Netherlands and 319 (33%) from Belgium (see supplemental material for participating centres and the number of included patients per centre). The majority of patients were included at the outpatient department (57.1%). A little over one third of patients were female (*n* = 345, 35.7%). Hypertension was the most frequently documented comorbidity with a prevalence of 49%. “Lone AF”, defined as the absence of any comorbidity in patients aged < 65 years, was present in 117 patients (12.1%).

Baseline characteristics of the total study population, with a comparison between the countries, are shown in Tab. 1. Noteworthy differences between the two countries, among others, are the higher prevalence of heart failure (21.4% vs 15.0%, *p* = 0.013), obstructive sleep apnoea (14.1% vs 4.1%, *p* < 0.001), type 2 diabetes mellitus (22.6% vs 15.5%, *p* = 0.007), and hyperlipidaemia (58.5% vs 36.8%, *p* < 0.001) in Belgian patients. Belgian patients also had a higher body mass index on average (29 ± 6 vs 28 ± 5, *p* < 0.001). In Belgium, more hospitalised patients were included in the registry (55.2% vs 36.9%, *p* < 0.001).

**Table 1:** Baseline characteristics of the study population with a comparison between the Netherlands and Belgium

Variable	NL & BE *N* = 967		The Netherlands *N* = 648		Belgium *N* = 319		NL vs BE
	*N* (%)	Missing	*N* (%)	Missing	*N* (%)	Missing	*p*-value
Female sex	345 (35.7)	–	231 (35.6)	–	114 (35.7)	–	0.978
Age	69.1 (±10.8)	–	68.7 (±10.9)	–	70.0 (±10.6)	–	0.072
BMI	28.3 (±5.3)	45	27.9 (±4.9)	40	29.2 (±5.9)	5	**<** **0.001**
Inpatient	415 (42.9)	–	239 (36.9)	–	176 (55.2)	–	**<** **0.001**
Outpatient	552 (57.1)		409 (63.1)		143 (44.8)		
*Type of AF*		6		3		3	** 0.001**
– First classified	92 (9.6)		65 (10.1)		27 (8.5)		
– Paroxysmal	341 (35.5)		256 (39.7)		85 (26.9)		
– Persistent	324 (33.7)		196 (30.4)		128 (40.5)		
– Long-standing pers	23 (2.4)		12 (1.9)		11 (3.5)		
– Permanent	181 (18.8)		116 (18.0)		65 (20.6)		
EHRA mean	1.8 (±0.8)	1	1.7 (±0.8)	–	1.8 (±0.8)	1	0.167
*EHRA*							0.365
– 1	448 (46.4)		308 (47.5)		140 (44.0)		
– 2	329 (34.1)		219 (33.8)		210 (34.6)		
– 3	171 (17.7)		112 (17.3)		59 (18.6)		
– 4	18 (1.9)		9 (1.4)		9 (2.8)		
Previous PVI	107 (11.1)	–	67 (10.3)	–	40 (12.5)	–	0.305
LVEF on echocardiogram	54.4 (±12.0)	367	53.8 (±11.6)	285	55.4 (±12.5)	82	0.098
LA diameter on echocardiogram	47.6 (±10.3)	414	46.7 (±10.6)	283	49.4 (±9.5)	131	** 0.003**
Lone AF	117 (12.1)	–	75 (11.6)	–	42 (13.2)	–	0.475
Hypertension	470 (49.0)	8	311 (48.6)	8	159 (49.8)	–	0.715
Coronary artery disease	218 (22.8)	12	152 (23.8)	9	66 (20.9)	3	0.315
Heart failure	165 (17.1)	1	97 (15.0)	–	68 (21.4)	1	** 0.013**
– NYHA I	51 (30.9)		33 (34)		18 (26.5)		
– NYHA II	72 (43.6)		50 (51.5)		22 (32.4)		
– NYHA III	37 (22.4)		12 (12.4)		25 (36.8)		
– NYHA IV	5 (3.0)		2 (2.1)		3 (4.4)		
Valvular heart disease	382 (40.5)	24	258 (41.3)	24	124 (38.9)	–	0.464
Dilated CMP	43 (4.5)	11	17 (2.7)	11	26 (8.2)	–	**<** **0.001**
Congenital heart disease	16 (1.7)	9	12 (1.9)	9	4 (1.3)	–	0.478
Hypertrophic CMP	33 (3.5)	11	10 (1.6)	10	23 (7.2)	1	**<** **0.001**
Pulmonary art. hypertension	41 (4.3)	13	3 (0.5)	9	38 (12.1)	4	**<** **0.001**
OSAS	67 (7.3)	44	26 (4.1)	16	41 (14.1)	28	**<** **0.001**
Peripheral vascular disease	77 (8.0)	5	47 (7.3)	3	30 (9.5)	2	0.242
T2DM	172 (17.9)	4	100 (15.5)	4	72 (22.6)	–	** 0.007**
Lipid disorder	416 (44.1)	24	231 (36.8)	21	185 (58.5)	3	**<** **0.001**
Hypothyroidism	29 (3.2)	8	7 (1.1)	4	22 (7.5)	4	**<** **0.001**
Hyperthyroidism	11 (1.2)	8	6 (1.0)	4	5 (1.6)	4	0.548
Prior thromboembolic event	155 (16.0)	–	99 (15.3)	–	56 (17.6)	–	0.364
Prior haemorrhagic event	52 (5.4)	1	30 (4.6)	1	22 (6.9)	–	0.143
LAA-occlusion	11 (1.2)	34	10 (1.6)	3	1 (0.3)	31	0.188
CHA_2_DS_2_-VASc mean	2.7 (±1.7)	3	2.6 (±1.7)	–	2.9 (±1.7)	3	** 0.010**
*CHA*_*2*_*DS*_*2*_*-VASc*							0.265
– 0	95 (9.9)		70 (10.8)		25 (7.9)		
– 1	153 (15.9)		109 (16.8)		44 (13.9)		
– 2	199 (20.6)		135 (20.8)		64 (20.3)		
– 3	210 (21.8)		139 (21.5)		71 (22.5)		
– 4	177 (18.4)		117 (18.1)		60 (19.0)		
– 5	70 (7.3)		45 (6.9)		25 (7.9)		
– 6	41 (4.3)		22 (3.4)		19 (6.0)		
– 7	13 (1.3)		9 (1.4)		4 (1.3)		
– 8	6 (0.6)		2 (0.3)		4 (1.3)		
HAS-BLED mean	1.5 (±1.0)	–	1.4 (±1.0)	–	1.6 (±1.0)	–	** 0.003**
*HAS-BLED*							** 0.011**
– 0	162 (16.8)		119 (18.4)		43 (13.5)		
– 1	357 (36.9)		252 (38.8)		105 (32.6)		
– 2	300 (31.0)		187 (28.9)		113 (35.4)		
– 3	123 (12.7)		76 (11.7)		47 (14.7)		
– 4	18 (1.9)		8 (1.2)		10 (3.1)		
– 5	7 (0.7)		6 (0.9)		1 (0.3)		
*Alcohol use*		296		240		56	0.686
– None	250 (37.3)		143 (35.0)		107 (40.7)		
– < 1 U/day	196 (29.2)		124 (30.4)		72 (27.4)		
– 1 U/day	93 (13.9)		59 (14.5)		34 (12.9)		
– 2–3 U/day	99 (14.8)		62 (15.2)		37 (14.1)		
– 4 or more	33 (4.9)		20 (4.9)		13 (4.9)		
*Smoking*		150		143		7	0.237
– No smoker	423 (51.8)		250 (49.5)		173 (55.4)		
– Currently	103 (12.6)		65 (12.9)		38 (12.2)		
– Former	291 (35.6)		190 (37.6)		101 (32.4)		

*AF* atrial fibrillation, *BE* Belgium, *BMI* body mass index, *CHA*_*2*_*DS*_*2*_*-VASc* congestive heart failure, hypertension, age ≥ 75 years [doubled], diabetes mellitus, prior stroke [doubled], vascular disease, age 65–74, sex category,* CMP* cardiomyopathy,* EHRA* European Heart Rhythm Association, *LA* left atrial, *LAA* left atrial appendage, *LVEF* left ventricular ejection fraction, *NL* the Netherlands, *NYHA* New York Heart Association, *OSAS* obstructive sleep apnoea syndrome, *T2DM* type 2 diabetes mellitus

Baseline laboratory measurements are shown in Supplementary Table 1. Belgian patients had on average a lower total cholesterol level (4.3 ± 1.2 vs 4.7 ± 1.2, *p* = 0.002), corresponding with the higher prescription rate of statins (46.9% vs 40.2%, *p* = 0.049, Tab. 2). Furthermore, N‑terminal pro-brain natriuretic peptide (NT-proBNP) levels were higher in Belgian patients (1714 [IQR 636–2803] vs 448 [IQR 228–1081], *p* = 0.018), in line with the higher amount of patients with heart failure (21.4% vs. 15.0%, *p* = 0.013, Tab. 1), even though the number of NT-proBNP measurements were low in Belgium and disproportionate between the two countries, 10 (3%) versus 119 (18%) patients.

**Table 2:** Baseline medication of the study population with a comparison between the Netherlands and Belgium

Medication	NL & BE *N* = 967		The Netherlands *N* = 648		Belgium *N* = 319		NL vs BE
	*N* (%)	Missing	*N* (%)	Missing	*N* (%)	Missing	*p*-value
VKA	453 (46.9)	2	344 (53.2)	1	109 (34.3)	1	**<** **0.001**
Rivaroxaban	188 (19.5)	2	124 (19.1)	1	64 (20.1)	1	0.723
Dabigatran	99 (10.3)	2	63 (9.7)	1	36 (11.3)	1	0.446
Apixaban	130 (13.5)	2	57 (8.8)	1	73 (23.0)	1	**<** **0.001**
Edoxaban	5 (0.5)	2	5 (0.8)	1	–	1	0.178
LMWH	13 (1.3)	2	1 (0.2)	1	12 (3.8)	1	**<** **0.001**
Any anticoagulation drug	887 (91.9)	2	593 (91.7)	1	294 (92.5)	1	0.669
ASA	101 (10.5)	2	40 (6.2)	1	61 (19.2)	1	**<** **0.001**
Clopidogrel	29 (3.0)	2	19 (2.9)	1	10 (3.1)	1	0.859
Ticagrelor	4 (0.4)	2	4 (0.6)	1	–	1	0.309
Dipyridamole	2 (0.2)	2	1 (0.2)	1	1 (0.3)	1	0.551
Sotalol	141 (14.6)	2	111 (17.2)	1	30 (9.4)	1	** 0.001**
– Mean ± SD dose per day in mg	155 ± 74		149 ± 73		176 ± 75		0.056
Amiodaron	128 (13.3)	3	38 (5.9)	2	90 (28.3)	1	**<** **0.001**
– Mean ± SD dose per day in mg	232 ± 105		208 ± 78		241 ± 115		0.052
Flecainide	80 (8.3)	2	53 (8.2)	1	27 (8.5)	1	0.874
– Mean ± SD dose per day in mg	151 ± 57		142 ± 54		169 ± 61		0.078
Propafenone	4 (0.4)	2	–	1	4 (1.3)	1	** 0.012**
Disopyramide	3 (0.3)	2	2 (0.3)	1	1 (0.3)	1	0.999
Any anti-arrhythmic drug	354 (36.7)	2	203 (31.4)	1	151 (47.5)	1	**<** **0.001**
Beta blocker	541 (56.1)	3	367 (56.8)	2	174 (54.7)	1	0.538
CCB	171 (17.7)	2	117 (18.1)	1	54 (17.0)	1	0.673
Digoxin	141 (14.6)	2	126 (19.5)	1	15 (4.7)	1	**<** **0.001**
Any rate control drug	653 (67.7)	2	448 (69.2)	1	205 (64.5)	1	0.136
ACE inhibitor	307 (31.8)	2	185 (28.6)	1	122 (38.4)	1	** 0.002**
Angiotensin receptor blocker	196 (20.3)	2	132 (20.4)	1	64 (20.1)	1	0.920
Aliskiren	6 (0.6%)	2	–	1	6 (1.9)	1	** 0.001**
Aldosterone blockers	65 (6.7)	2	35 (5.4)	1	30 (9.4)	1	** 0.019**
Any RAAS inhibiting drug	529 (54.8)	2	327 (50.5)	1	202 (63.5)	1	**<** **0.001**
Diuretics	335 (34.7)	2	221 (34.2)	1	114 (35.8)	1	0.604
Non-DHP CCB	58 (6.0)	2	46 (7.1)	1	12 (3.8)	1	** 0.040**
Statin	409 (42.4)	2	260 (40.2)	1	149 (46.9)	1	** 0.049**
Oral anti-diabetics	133 (13.8)	2	73 (11.3)	1	60 (18.9)	1	** 0.001**
Insulin	35 (3.6)	2	24 (3.7)	1	11 (3.5)	1	0.845
Thyroid hormones	64 (6.6)	2	19 (2.9)	1	45 (14.2)	1	**<** **0.001**
Thyroid-suppressing drugs	10 (1.0)	2	7 (1.1)	1	3 (0.9)	1	0.842
Proton pump inhibitors	275 (28.5)	2	201 (31.1)	1	74 (23.3)	1	** 0.012**

*ACE* angiotensin-converting enzyme, *ASA* acetylsalicylic acid, *CCB* calcium channel blocker, *DHP* dihydropyridine, *LMWH* low molecular weight heparins, *RAAS* renin-angiotensin-aldosterone system, *SD* standard deviation, *VKA* vitamin K antagonist

### Rate versus rhythm control therapy

At baseline, the majority of the patients were on a rhythm control strategy according to the treating physician (*n* = 565, 58.6%), whereas 326 patients (33.8%) were on a rate control strategy. For the remainder of the patients (*n* = 73, 7.6%), no decision regarding the treatment strategy had been made yet.

In total, 354 patients (36.7%) were prescribed an anti-arrhythmic drug (AAD) for daily use and 653 (67.7%) a rate control drug (Tab. 2). Belgian patients were more frequently on AAD therapy (47.5% vs 31.4%, *p* < 0.001) with significantly more amiodarone users compared with their Dutch counterparts (28.3% vs 5.9%, *p* < 0.001), whereas Dutch patients more often received sotalol (17.2% vs 9.4%, *p* = 0.001). The prescription rates for the other AADs were comparable (Tab. 2). For rate control drugs, only digoxin use was significantly different between the two countries, with more frequent prescription in the Netherlands (19.5% vs 4.7%, *p* < 0.001).

Of the 565 patients with rhythm control as treatment strategy, 313 patients (55.4%) were on daily AAD treatment. In the remainder of patients, 14 (5.6%) were planned for electrical cardioversion and 1 (0.4%) for surgical AF ablation, meaning that 237 patients (41.9%) were on a rhythm control strategy without daily AAD use or a planned rhythm intervention.

Of the 326 patients with rate control as treatment strategy, 280 patients (86.2%) were on rate control drugs, whereas 45 (13.8%) were not (for 1 patient medication was missing). Notably, 34 of the 326 patients with rate control as treatment strategy (10.4%) were on daily AAD treatment. In the remainder of patients, 4 (1.4%) were planned for electrical cardioversion, meaning that 38 patients (11.8%) were wrongfully classified as rate control and should in fact have been classified as rhythm control regarding the treatment strategy.

### Anticoagulant therapy

**Fig. 1:**
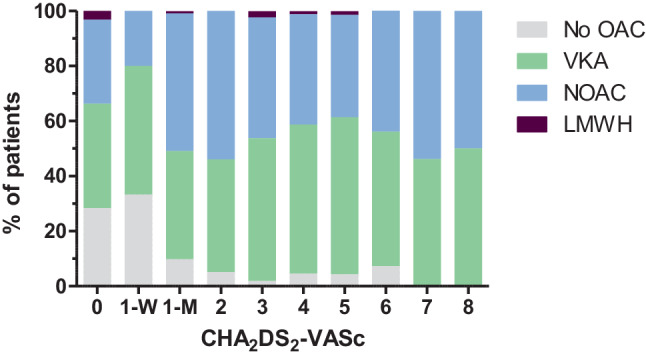
Stacked bar chart showing the percentages of patients with and without anticoagulation per CHA_2_DS_2_-VASc score, also specifying the type of anticoagulant. CHA_2_DS_2_-VASc 1 is further subdivided by sex. *CHA*_*2*_*DS*_*2*_*-VASc* congestive heart failure, hypertension, age ≥ 75 years [doubled], diabetes mellitus, prior stroke [doubled], vascular disease, age 65–74, sex category,* LMWH* low molecular weight heparins, *M* men, *NOAC* non-vitamin K antagonist oral anticoagulants, *OAC* oral anticoagulation, *VKA* vitamin K antagonists, *W* women

**Fig. 2:**
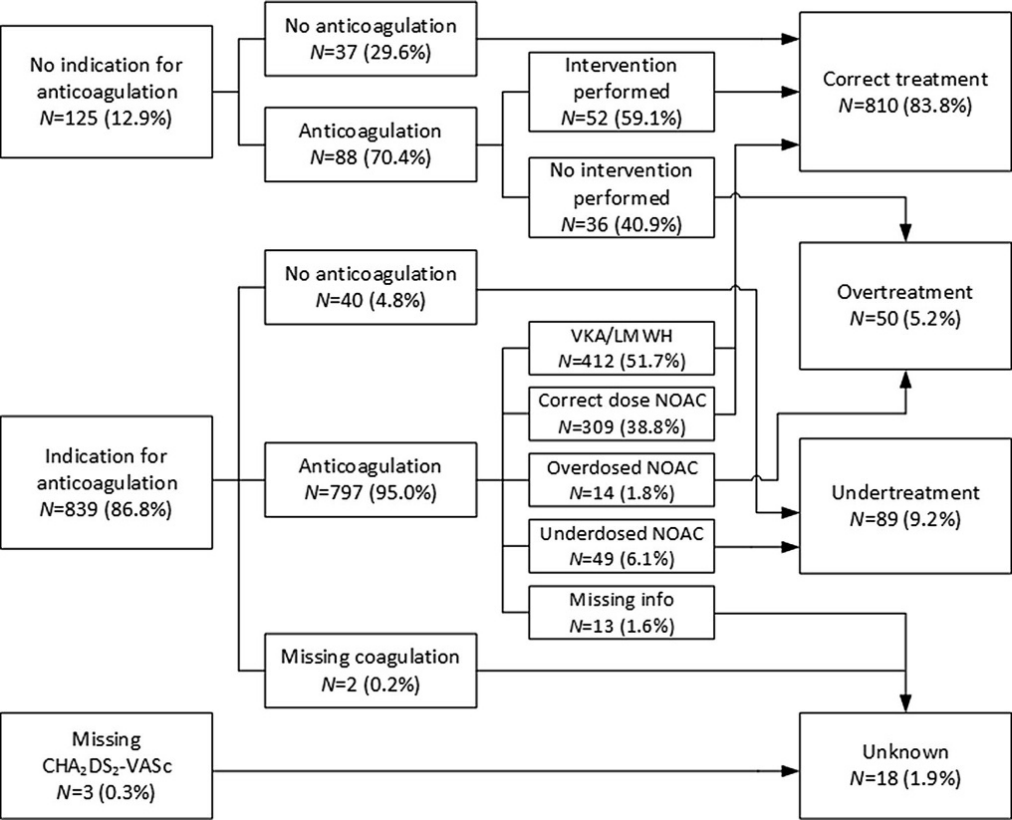
Flowchart for the appropriateness of anticoagulant treatment for the whole population. *LMWH* low molecular weight heparins, *NOAC* non-vitamin K antagonist oral anticoagulants, *VKA* vitamin K antagonists

At baseline, 887 patients (91.9%) received anticoagulant treatment, with a larger proportion of patients using a non-vitamin K antagonist oral anticoagulant (NOAC) in Belgium than in the Netherlands (54.4% vs 38.4%). Fig. 1 depicts the percentage of patients with and without anticoagulation for the different CHA_2_DS_2_-VASc (congestive heart failure, hypertension, age ≥ 75 years [doubled], diabetes mellitus, prior stroke [doubled], vascular disease, age 65–74, sex category) scores, also specifying the type of anticoagulant. For men with CHA_2_DS_2_-VASc 0 and for women with CHA_2_DS_2_-VASc 1, the anticoagulation rates were 71.6% (68/95 patients) and 66.7% (20/30 patients) respectively. In these patients with anticoagulation, a rhythm intervention was performed during the baseline assessment or planned for the near future in 45 men (66.2%) and 7 women (35%). The remaining 23 men (33.8%) and 13 women (65%) were on anticoagulant treatment without a clear indication (Fig. 2).

For patients with NOACs, over- and underdosing of NOACs were checked according to the dose reduction criteria for the respective drug [6]. In total, 14 patients were prescribed the full dose for a NOAC, whereas they should have received the reduced dose according to the Summary of Product Characteristics (SmPC). This was the case for 12/148 patients with rivaroxaban and 2/57 with dabigatran (“Overdosed NOAC” in Fig. 2). No overdosing was present for both apixaban (*n* = 70) and edoxaban (*n* = 5). Underdosing occurred in 49 patients (“Underdosed NOAC” in Fig. 2), i.e. 38/50 patients with apixaban, 8/24 with rivaroxaban, and 3/30 with dabigatran. Reduced dose of edoxaban was not prescribed. In total, 63 (16.7%) of 372 patients were not correctly dosed.

#### Follow-up

One year follow-up data was available in 882 (91.2%) of 967 patients. MACCE occurred in 37 (4.2%) of these patients. In total, 27 (3.1%) patients died within a year, 8 of whom from a cardiovascular cause (0.9%). Ischaemic stroke occurred in 7 patients (0.8%) and TIA in 3 (0.3%). Two patients (0.2%) had intracranial haemorrhage, one of whom died as a consequence, whereas 14 patients (1.6%) had a major extracranial bleeding. Myocardial infarction occurred in 5 patients (0.6%), one of these patients also had an ischaemic stroke.

Of the patients with ischaemic stroke/TIA, 7 out of 10 were on anticoagulant therapy at baseline. The remaining 3 patients were not, despite a CHA_2_DS_2_-VASc score of 2, 3 and 5 respectively. However, at follow-up all 3 patients were on anticoagulant therapy, but 1 of the other 7 patients with an ischaemic event had stopped using anticoagulant therapy. As for the 16 patients with a major bleeding event during follow-up, they all had anticoagulant therapy at baseline. At follow-up, 1 patient had stopped therapy (6.3%), 14 patients were still on anticoagulant therapy, and 1 had died as a consequence of the intracranial haemorrhage. Of the 37 patients with MACCE during follow-up, only 1 was underdosed (major extracranial bleeding event), totalling to 4 patients (10.8%) with undertreatment. No overtreatment was seen in these patients.

During follow-up, the most frequently established new diagnosis was heart failure (40 patients, 4.5%), followed by chronic kidney disease, defined as initiation of chronic dialysis, renal transplantation, or serum creatinine ≥ 200 μmol/l (2.26 mg/dl), in 23 patients (2.6%), and hypertension in 16 patients (1.8%). In addition, new-onset overt coronary artery disease was diagnosed in 15 patients (1.5%), diabetes mellitus in 8 (0.9%), and peripheral artery disease in 4 (0.5%) patients.

### Rhythm follow-up and interventions

Rhythm follow-up was available in 839 patients (86.8%). AF progression and AF regression occurred at a similar rate, with 57 (17.3%) of 329 patients with paroxysmal AF at baseline progressing into persistent or permanent AF after one year of follow-up and 87 (17.6%) of 494 patients with persistent or permanent AF regressing into paroxysmal AF. Of the regressors, 38 patients (43.7%) were already on AAD treatment at baseline. A rhythm intervention was performed during follow-up in 20 (23.0%) of 87 patients showing regression, i.e. in 12 patients (13.8%) an AAD was initiated, in 9 patients (10.3%) a catheter ablation for AF was performed (1 of whom also started AAD therapy), and in 2 patients (2.3%) surgery for atrial fibrillation was performed (both of them started AAD therapy as well). The remaining 67 patients (77%) showed regression in type of AF without rhythm intervention.

In total, 233 (26.4%) of 882 patients had at least 1 rhythm intervention during follow-up, i.e. 155 patients (17.6%) underwent electrical cardioversion, 33 patients (3.7%) pharmacological cardioversion, and 77 patients (8.7%) catheter ablation for AF. Of the latter, 72 patients underwent a pulmonary vein isolation and 7 atrioventricular node ablation with pacemaker implantation. Furthermore, 14 patients (1.6%) underwent surgery for AF, 9 of which as stand-alone procedure and 5 concomitantly with another intervention, and 19 patients (2.2%) underwent an ablation for atrial flutter, 6 of which as stand-alone and 13 concomitantly with AF ablation.

Supplementary Fig. 1 shows the changes in treatment strategy for AF from baseline to follow-up. Relatively more patients changed from rate to rhythm control therapy than vice versa (24% vs 20%), although absolute numbers show the opposite (66 vs 99 patients). The majority of patients were on the same treatment strategy at baseline and at 1‑year follow-up, i.e. 610 of 775 patients (79%).

## Discussion

In this analysis of the EORP-AF LTG registry, we provide a contemporary representation of the characteristics, treatment, and outcomes of AF patients in the Netherlands and Belgium, both inpatient and outpatient. Overall, hypertension was the most frequently documented comorbidity and present in almost half of all patients. “Lone AF” was present in 12% of the patients, which is comparable with the 10% seen in the Euro Heart Survey 15 years ago [7].

Strikingly, a large proportion of the patients without an indication for chronic anticoagulant treatment, i.e. men with a CHA_2_DS_2_-VASc score of 0 and women with a score of 1, are in fact anticoagulated at baseline (70%). A slim majority (59%) of these patients underwent a rhythm intervention at or shortly after baseline, resulting in overtreatment of patients with anticoagulant therapy in the remainder, which translates into 5% of the total population being overtreated (Fig. 2). Undertreatment of patients was even more prevalent, with 9% of the patients either receiving no anticoagulation at all in the presence of an indication based on the CHA_2_DS_2_-VASc score, or receiving the reduced dose of a NOAC without a reason for dose reduction. In total, 17% of the patients using a NOAC are incorrectly dosed. Previous reports have shown higher rates, with a Belgian single-centre study showing 25% and a Turkish single-centre study showing 37% of inappropriate dosing [8, 9].

MACCE occurred in 37 patients (4.2%) after 1 year of follow-up, which is relatively low. To put into perspective, 208 (5.3%) of 3890 patients in the Euro Heart Survey died of cardiovascular cause or developed ischaemic stroke/TIA after one year, not taking into account major bleeding events and myocardial infarction, which comprise half of the MACCE in this registry [10]. Given the rather low rate of ischaemic complications and major bleeding events after one year in this population, it is hard to draw any conclusions regarding the relationship between these endpoints and undertreatment or overtreatment. All patients with a major bleeding event were anticoagulated with correct dosing, whereas in the 10 patients with ischaemic stroke/TIA, 7 were correctly treated and 3 were incorrectly not anticoagulated. The rates of ischaemic stroke/TIA in patients with and without anticoagulants are in line with previous reports, i.e. 3.75% (3 of 80) in those without and 0.79% (7 of 887) in those with anticoagulants [11].

Rhythm control was the preferred treatment strategy at baseline in 59% of the analysed patients, whereas one third of the patients were on a rate control strategy. The majority of patients (80%) were on the same treatment strategy at baseline and at follow-up. Misclassification regarding treatment strategy was observed in 12% of patients. In these cases, rate control was the appointed treatment strategy, but patients were either receiving AAD treatment on a daily basis or were scheduled for electrical cardioversion. Therefore, more awareness regarding definitions and the correct use of terminology is warranted [6]. On a side note, in patients in whom rate control drugs do not sufficiently achieve adequate rate control physicians may feel the need to use AAD for rate control. Information regarding the reasoning behind the treatment strategy was not reported, so this could not be assessed in this population.

Both AF progression and regression occurred at a similar rate of 17% per year, keeping in mind that a misclassification in type of AF inherently leads to a misclassification in AF progression and regression. In a quarter of the regressors, AAD treatment was initiated or a rhythm intervention was performed during follow-up. The remainder of the patients regressed without a change in rhythm therapy, arising the question whether misclassification also plays a role in this high rate of regression after 1 year. The only way to solve this issue, is by increasing the monitoring duration, e.g. performing more 24-hour Holter recordings or making use of more rigorous modalities such as implantable loop recorders [12].

Some differences between the two countries are present at baseline, e.g. the higher prevalence of heart failure, type 2 diabetes, and sleep apnoea in Belgium. This is most likely caused by the higher proportion of hospitalised patients included in Belgium compared with the Netherlands (55% vs 37%). In addition to these differences in patient characteristics, distinct drug prescription patterns are discernible between the two countries, i.e. amiodarone is more frequently used in Belgium and sotalol more frequently in the Netherlands. No differences are seen in the use of flecainide, whereas propafenone is only prescribed in Belgium. In addition, digoxin use is more prevalent in the Netherlands for rate control. Regarding anticoagulant therapy, Belgian patients were more frequently on NOACs compared with their Dutch counterparts (54% vs 38%), showing the faster implementation and uptake of NOACs in Belgium, as shown previously [13].

### Strengths and limitations

As in all registries, the strength of this registry is dependent upon the completeness of the data obtained by all participating centres, so clinical factors can be both over- and underrepresented. Furthermore, the reported data are observational. In order to reduce selection bias, participating centres were asked to include consecutive AF patients. Nonetheless, we believe the data presented in this article are comprehensive and provide a representative overview of AF patients in the Netherlands and Belgium and the treatment they receive.

Of note, patients were deemed underdosed for dabigatran only if both the regular and the lenient dose reduction criteria, i.e. age 75–80 years and eGFR 30–50 ml/min for which dose reduction can be considered, were not met. In addition, verapamil/diltiazem shared the same checkbox in the case report file, so it is unclear which of the two the patient is using, therefore we cannot be sure whether the dose reduction is adequate. Furthermore, treating physicians may have chosen to treat patients at increased risk of bleeding with the reduced dose of dabigatran, since the reduced dose is non-inferior to vitamin K antagonists (VKAs), this was not taken into account. For apixaban, the rate of underdosing is most likely to be overestimated, since in some patients not the daily dose of apixaban was filled in the case report file as requested, but rather the dose for a single gift. In patients with 10 mg and 2.5 mg of apixaban, it is clear whether the patient receives the reduced or full dose, for 5 mg however, this is not clear. These patients were assessed as having the reduced dose of apixaban, meaning that the rate of underdosing might be overestimated as these patients could in fact be on twice daily 5 mg of apixaban.

## Conclusion

Overtreatment and undertreatment with anticoagulants are still observable in 14% of this contemporary, West-European AF population. Still, MACCE occurred in only 4% of the patients after 1 year of follow-up.

## Supplementary Information

Supplementary Tables 1–3

**Supplementary Fig. 1** Sankey plot showing the treatment strategy for atrial fibrillation at baseline and follow-up and the changes occurred during follow-up.
