# Supplementary material for: Contemporary management of patients with atrial fibrillation in the Netherlands and Belgium: a report from the EORP-AF long-term general registry
Source: Neth Heart J. 2021 Sep 15;29(11):584–94. doi: 10.1007/s12471-021-01634-y (PMC8556427; doi:10.1007/s12471-021-01634-y)
Supplement: Supplementary file 1 — Supplementary Tables 1–3 [file 12471_2021_1634_MOESM1_ESM.docx]

Supplemental material

Contemporary management of atrial fibrillation patients in the Netherlands and Belgium: a report from the EORP-AF long-term general registry

**Supplementary Table 1.** Baseline laboratory measurements of the study population with a comparison between the Netherlands and Belgium.

| **Variable** | **Netherlands & Belgium**  ***N*=967** | | | **the Netherlands**  ***N*=648** | | | **Belgium**  ***N*=319** | | | **NL vs BE**  ***p*-value** |
| --- | --- | --- | --- | --- | --- | --- | --- | --- | --- | --- |
|  | **Mean (±SD)** | **Median (IQR)** | ***N*** | **Mean (±SD)** | **Median (IQR)** | ***N*** | **Mean (±SD)** | **Median (IQR)** | ***N*** |  |
| Creatinine, in umol/l | 94 (±36) | 87 (74-104) | 870 | 93 (±34) | 87 (75-102) | 584 | 96 (±39) | 88 (72-107) | 286 | 0.275 |
| Hb, in mmol/l | 8.8 (±1.1) | 8.9 (8.1-9.5) | 808 | 8.8 (±1.1) | 8.9 (8.1-9.5) | 524 | 8.7 (±1.3) | 8.8 (8.1-9.5) | 284 | 0.083 |
| TSH | 2.1 (±1.5) | 1.8 (1.2-2.8) | 630 | 2.3 (±1.4) | 1.9 (1.3-2.9) | 398 | 1.9 (±1.6) | 1.5 (0.9-2.4) | 232 | **0.001** |
| Total cholesterol,  in mmol/l | 4.6 (±1.2) | 4.5 (3.7-5.5) | 419 | 4.7 (±1.2) | 4.7 (4.0-5.5) | 289 | 4.3 (±1.2) | 4.2 (3.5-5.2) | 130 | **0.002** |
| HbA1c, in % | 6.3 (±1.2) | 6.0 (5.5-6.6) | 215 | 6.1 (±1.0) | 5.9 (5.4-6.5) | 149 | 6.5 (±1.6) | 6.1 (5.6-6.8) | 66 | 0.133 |
| NT-proBNP, in pg/ml | 1085 (±1509) | 481 (241-1217) | 129 | 1034 (±1533) | 448 (228-1081) | 119 | 1694 (±1063) | 1714 (636-2803) | 10 | **0.018** |
| BNP, in pg/ml | 407 (±278) | 355 (147-620) | 26 | 398 (±229) | 380 (213-512) | 17 | 422 (±368) | 222 (113-709) | 9 | 0.597 |

*BE* Belgium, *BNP* brain natriuretic peptide, *Hb* haemoglobin, *HbA1c* haemoglobin A1c, *NL* the Netherlands, *IQR* interquartile range, *TSH* thyroid stimulating hormone, *NT-proBNP* N-terminal pro-brain natriuretic peptide

**Supplementary Table 2.** Participating centres of the EORP-AF Long-Term General Registry in the Netherlands and included patients per centre.

| **Participating centre, city** | **Number of patients** |
| --- | --- |
| Maastricht University Medical Center +, Maastricht | 270 |
| Jeroen Bosch Hospital, ‘s Hertogenbosch | 144 |
| Medisch Spectrum Twente, Enschede | 83 |
| Academic Medical Center, Amsterdam | 50 |
| VieCuri Medical Center, Venlo | 40 |
| Tjongerschans Hospital, Heerenveen | 23 |
| Ziekenhuis Groep Twente, Hengelo | 20 |
| Flevo Hospital, Almere | 10 |
| Ommelander Hospital Group, Delfzijl | 6 |
| Erasmus Medical Center, Rotterdam | 2 |
| **Total** | 648 |

**Supplementary Table 3.** Participating centres of the EORP-AF long-term general registry in Belgium and included patients per centre.

| **Participating centre, city** | **Number of patients** |
| --- | --- |
| CHU Mont-Godinne UCL, Yvoir | 105 |
| DHU, Liège | 38 |
| Jessa Hospital, Hasselt | 30 |
| CHU Tivoli, La Louvière | 29 |
| CHR de la Citadelle, Liege | 25 |
| Hôpital Sainte Thérèse, Bastogne | 21 |
| CHC St. Joseph, Liege | 20 |
| Hôpital Saint-Joseph, Gilly | 20 |
| AZ Groningen | 12 |
| CHC Clinique Notre-Dame, Waremme | 8 |
| Maria Hospital, Overpelt | 7 |
| Cabinet de Cardiologie dr. P. Melon, Liege | 4 |
| **Total** | 319 |
