# Supplementary figures and images for: Contemporary management of patients with atrial fibrillation in the Netherlands and Belgium: a report from the EORP-AF long-term general registry
Source: Neth Heart J. 2021 Sep 15;29(11):584–94. doi: 10.1007/s12471-021-01634-y (PMC8556427; doi:10.1007/s12471-021-01634-y)

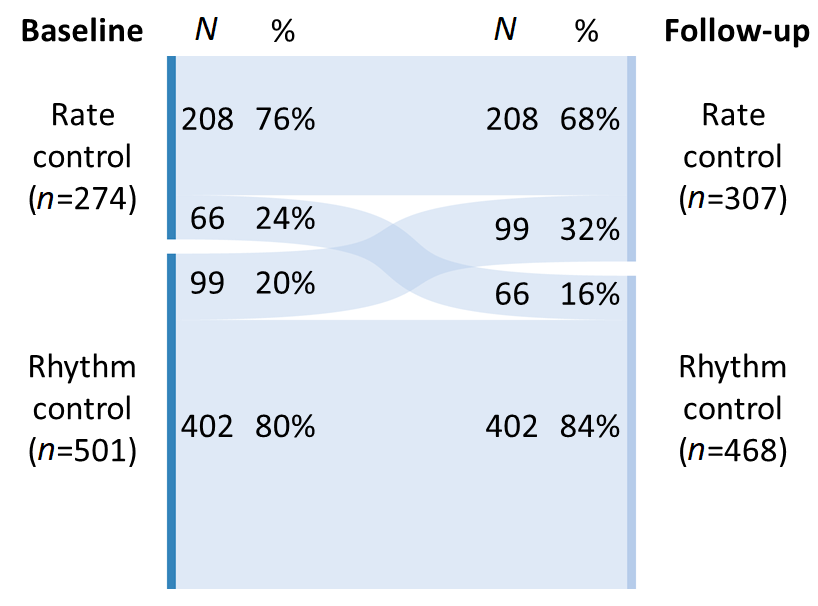

Supplement: Supplementary file 2 — Supplementary Fig. 1 Sankey plot showing the treatment strategy for atrial fibrillation at baseline and follow-up and the changes occurred during follow-up. [file 12471_2021_1634_MOESM2_ESM.png]
